# Supplementary material for: The nuclear lamina couples mechanical forces to cell fate in the preimplantation embryo via actin organization
Source: Nat Commun. 2023 May 29;14:3101. doi: 10.1038/s41467-023-38770-5 (PMC10226985; doi:10.1038/s41467-023-38770-5)
Supplement: Supplementary file 1 — Supplementary Information [file 41467_2023_38770_MOESM1_ESM.pdf]

Supplementary Figures

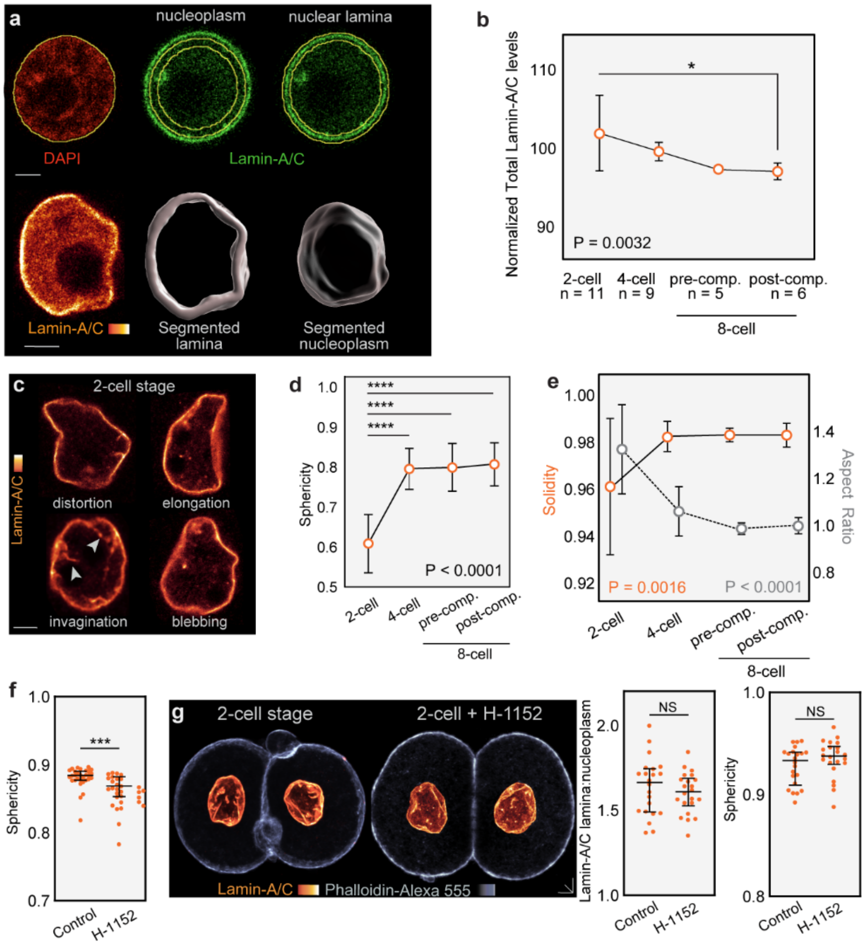

Supplementary Fig 1. Further characterization of Lamin-A in the embryo.

a, Example of computational segmentation of the nuclear lamina and nucleoplasm for quantification of Lamin-A/C levels in each subnuclear compartment. **b**, Quantification of total Lamin-A/C levels within the nucleus (combining both lamina and nucleoplasm compartments) at multiple developmental stages. n=11 for 2-cell, n=9 for 4-cell, n=5 pre-compaction, n=6 post-compaction, P=0.0032, Mann-Whitney U test. **c**, Nuclear morphology at the 2-cell stage is non-spherical, featuring distortion, invagination, elongation and blebbing. **d, e,** Nuclear sphericity and solidity increases from the 2-cell to 8-cell stage, while aspect ratio decreases. Dots represent the mean and error bars represent SD. n=11 for 2-cell, n=9 for 4-cell, n=5 pre-compaction. **f,** Nuclear sphericity decreases after treatment with H-1152 at the 8-cell stage. n=17 for control and H-1152. ***P=0.0008, Mann-Whitney U test. **g**, Nuclear sphericity and Lamin-A/C L:N shows no change after treatment with H-1152 at the 2-cell stage. n=22 for control and H-1152. NS p=0.26 for Lamin-A/C levels, p=0.226 for sphericity, Mann-Whitney U test. All statistical tests are two-tailed. Bars on dot plots represent median and interquartile range. Scale bars, 10 µm.

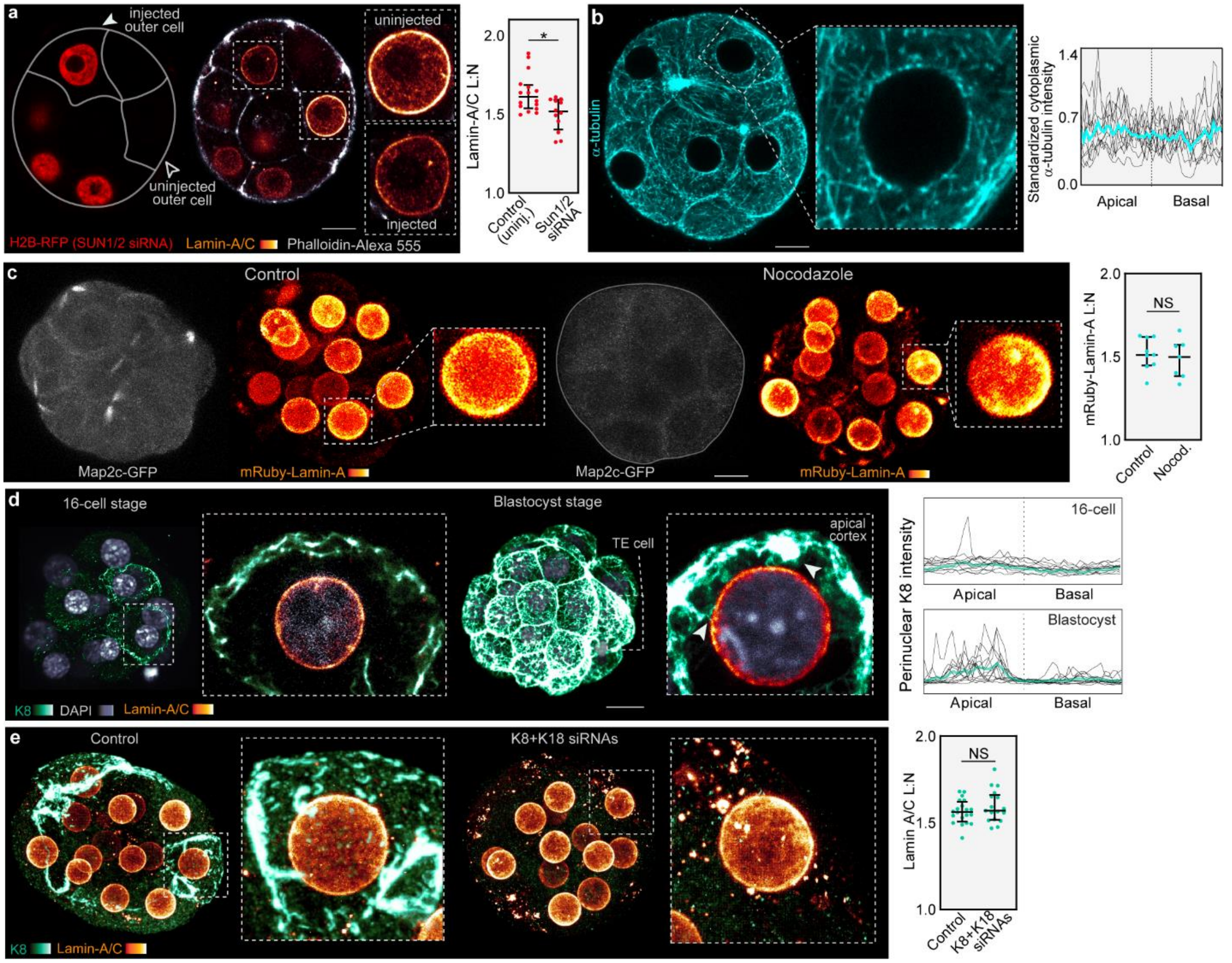

Supplementary Fig 2. Microtubules and keratin filaments do not regulate Lamin-A levels during inner-outer segregation

a, Knockdown of SUN1 and SUN2 by siRNA causes a decrease in Lamin-A/C levels. siRNAs were microinjected into a single cell of the 2-cell embryo. H2B-RFP was co-injected to label injected cells (arrowheads). n=16 for control, n=12 for Lamin-A siRNA. *P = 0.024, Mann-Whitney U test. **b**, Immunostaining for α–tubulin reveals microtubules throughout the cytoplasm, extending from the cortex to the nuclear lamina. Quantification of α–tubulin levels shows homogenous levels throughout the cytoplasm and perinuclear space. **c**, Treatment with nocodazole causes no change in Lamin-A/C L:N, as characterized by immunofluorescence. n=9 for control, n=7 for nocodazole. NS=0.607, Mann-Whitney U test. **d**, Embryos immunostained for Keratin-8 at the 16-cell and blastocyst stage show that dense networks of keratin filaments are only established later in development, after inner and outer cell identity is established. Keratin filaments are confined to the cortex at the 16-cell stage and are not detected in the cortex to lamina space until the blastocyst stage. Quantification shows Keratin-8 levels in the perinuclear space. **e**, Keratin network disruption following injection of siRNAs for K8+K18 does not disrupt Lamin-A/C L:N at the 16-cell stage. n=20 for control, n=18 for Krt siRNA NS P=0.612, Mann-Whitney U test. All statistical tests are two-tailed. Bars on dot plots represent median and interquartile range. Scale bars, 10 µm.

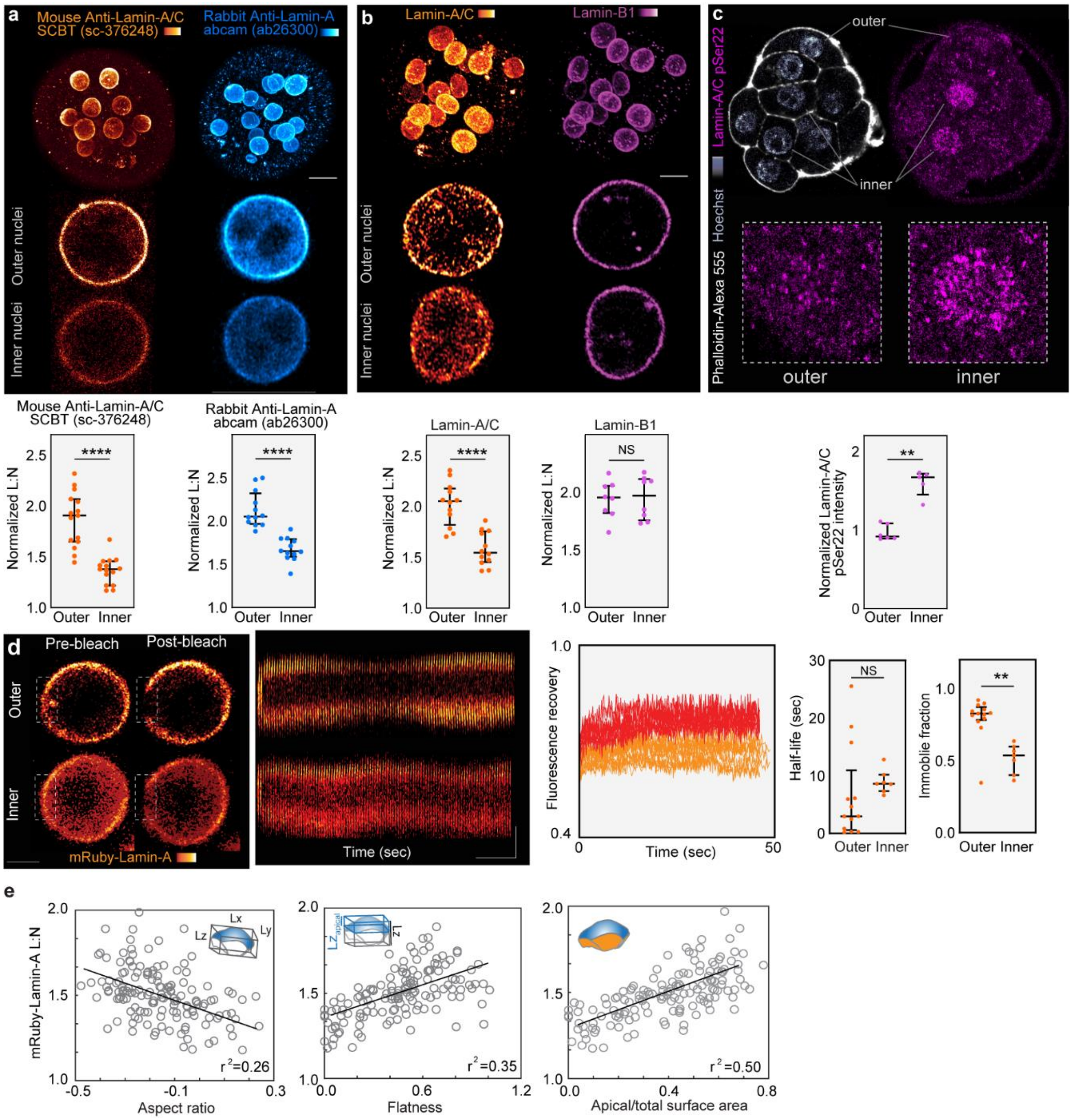

Supplementary Fig 3. Additional characterization of Lamin-A and dynamics during lineage segregation

a, Lamin-A L:N is differentially regulated between inner and outer cell as confirmed by immunofluorescence using two different antibodies. For sc-376248 n=15 for outer and inner. For ab26300 n=12 for outer and inner. ****P<0.0001, Mann-Whitney U test. **b**, Lamin-B L:N is not differentially regulated between inner and outer cells, as shown by immunofluorescence co-staining for Lamin-A/C and Lamin-B. Insets shows the same nuclei co-stained for both Lamin-A/C and Lamin-B. n=12 for Lamin-A/C outer and inner, n=8 for Lamin-B outer and inner, ****P<0.0001, NS P=0.763, Mann-Whitney U test. **c**, Lamin-A pSer22 levels are higher in inner cells, as shown by immunofluorescence using a Lamin-A/C Ser22 phospho-specific antibody. n=6 for outer, n=5 for inner. **P<0.0043, Mann-Whitney U test. **d**, FRAP experiments performed using mRuby-Lamin-A in outer

and inner cells of 16 cell embryos show that outer cells have a larger immobile fraction of mRuby-Lamin-A than inner cells, with no differences in the recovery time. Nuclei pre- and post-FRAP are shown with photobleached regions of interest and kymographs. Graphs show fluorescence recovery of over time for outer and inner cells. **P=0.0012, Mann-Whitney U test. **e**, Analysis of geometrical parameters in embryos co-expressing mRuby-Lamin-A and Utrophin-GFP reveals a tight correlation between mRuby-Lamin-A L:N and apical/total surface area. Lamin-A L:N also correlates with cell volume, aspect ratio and flatness. All statistical tests are two-tailed. Bars in dot plots represent median and interquartile range. Scale bars, 10 µm.

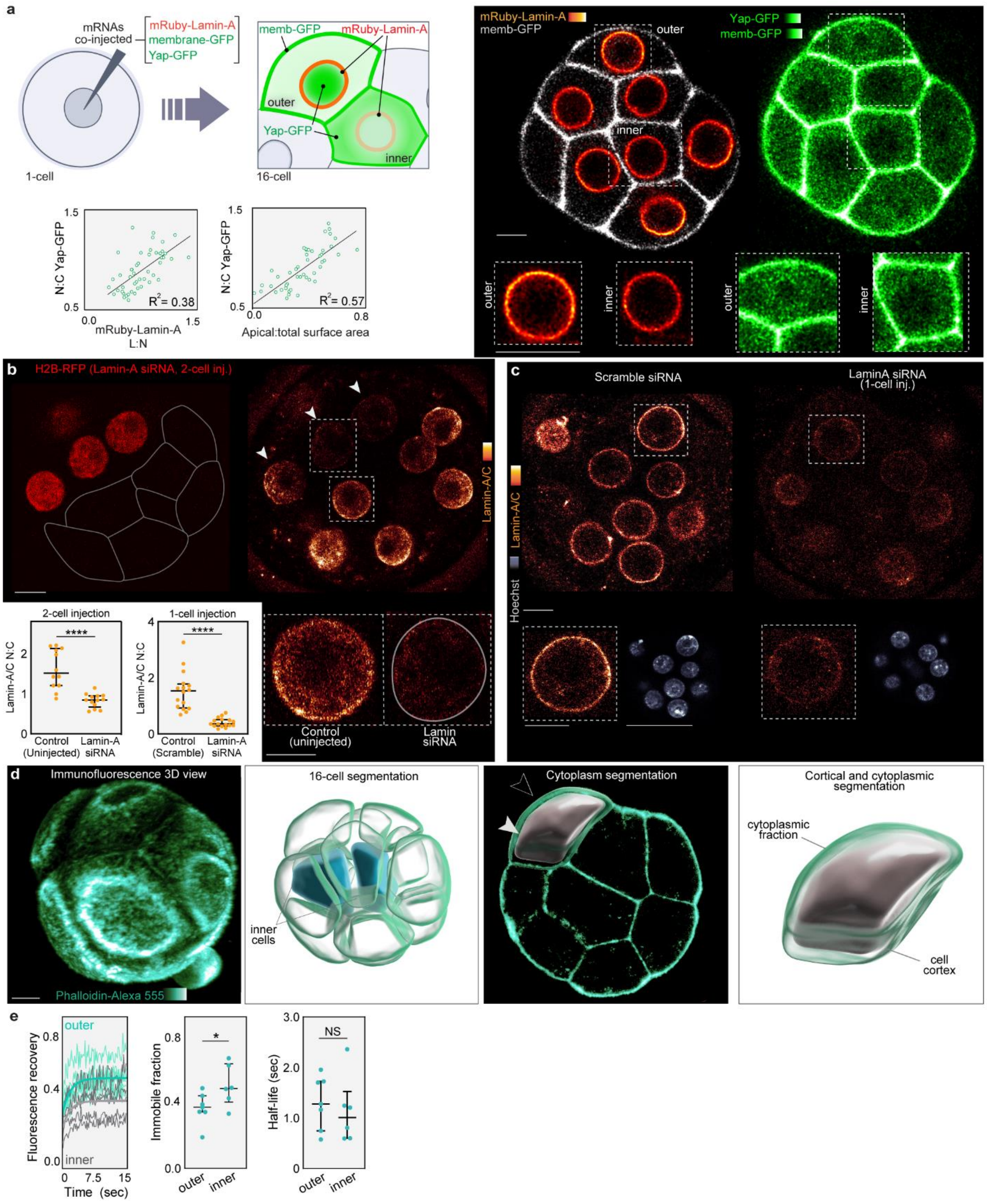

Supplementary Fig 4. Additional characterization of Lamin-A disruption and cell fate

a, Scheme shows microinjection strategy. Live-imaging of embryos co-expressing mRuby-Lamin-A, membrane-GFP and Yap-GFP show a strong correlation between

mRuby-Lamin-A L:N and the nuclear:cytoplasmic levels of Yap-GFP. **b, c**, Lamin-A siRNAs injected into a single cell at the two-cell stage (**b**) and at the 1-cell stage (**c**) show robust knockdown of Lamin-A/C expression validated by immunofluorescence. n=12 for 2-cell control, n=11 for 2-cell Lamin-A siRNA, n=16 for 1-cell control and Lamin-A siRNA, ****P<0.0001, Mann-Whitney U test. **d**, Example of 3D segmentation of cytoplasm (opaque) and cell cortex (transparent) performed in an embryo following phalloidin staining. This approach also allows inner (blue) and outer (green) cells to be computationally distinguished. **e**, FRAP performed using Emerald-Amot shows inner cells have a larger immobile fraction of Amot in the cytoplasm than outer cells. n = 6 for inner cells, n = 7 for outer cells. *P = 0.022, Mann-Whitney U test. All statistical tests are two-tailed. Bars in dot plots represent median and interquartile range. Scale bars, 10 µm.

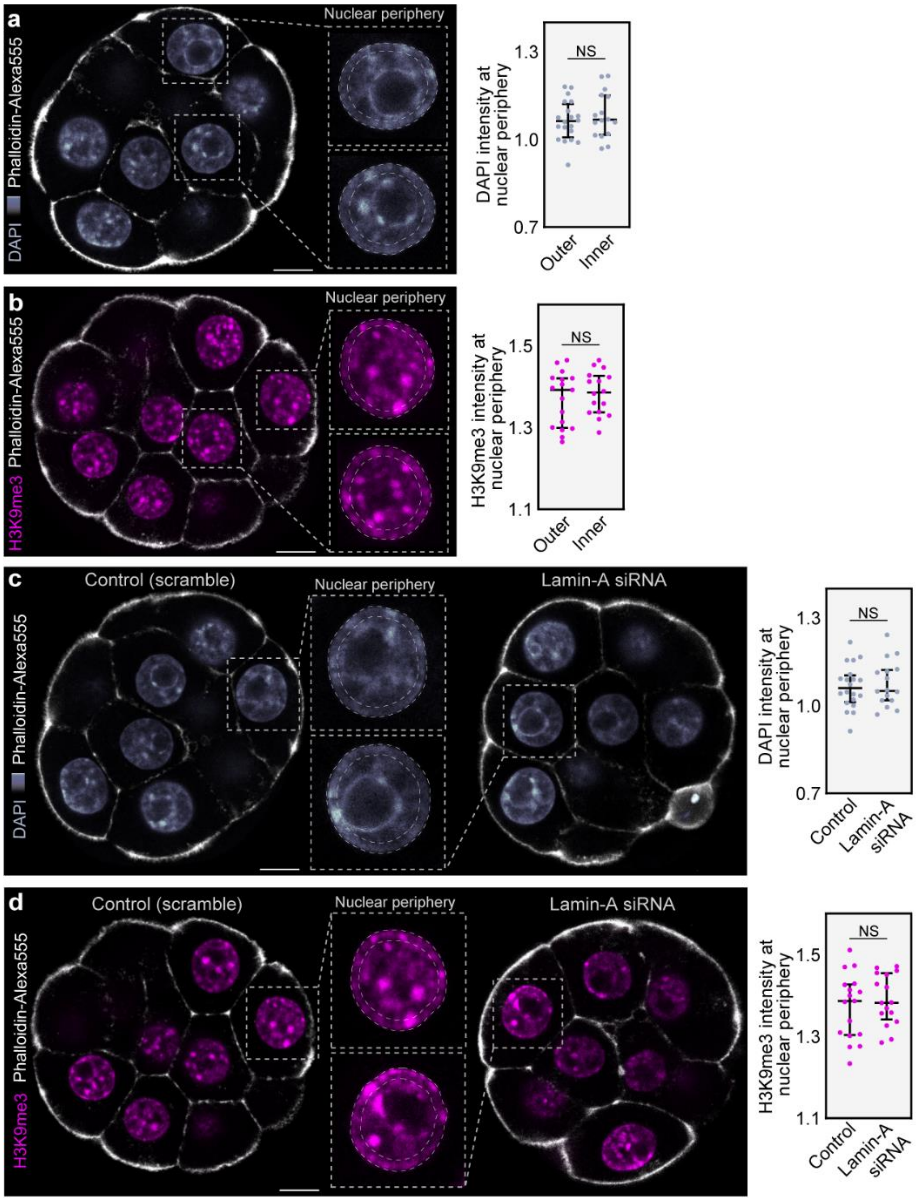

Supplementary Fig 5. Changes in Lamin-A localization during inner-outer segregation does not affect chromatin organization

a, b, Embryos at the 16-cell stage fixed and stained with DAPI (**a**) and immunostained for H3K9me3 (**b**) show no differences in organization at the nuclear periphery between inner and outer cells. n=16 for both experiments. NS=0.609 for DAPI and NS=0.656 for H3K9me3, Mann-Whitney U test **c, d,** Embryos injected with Lamin-A siRNA or scramble control and fixed at the 16-cell stage. Embryos were stained with DAPI (**c**) and immunostained for H3K9me3 (**d**) showing no differences in chromatin organization at the nuclear periphery between the two groups. n=20 for DAPI control and n=16 for DAPI Lamin-A siRNA. n=17 for H3K9me3 control and n=16 for H3K9me3 Lamin-A siRNA. NS=0.671 for DAPI and NS=0.557 for H3K9me3, Mann-Whitney U test. All statistical tests are two-tailed. Bars in dot plots represent median and interquartile range. Scale bars, 10 µm.

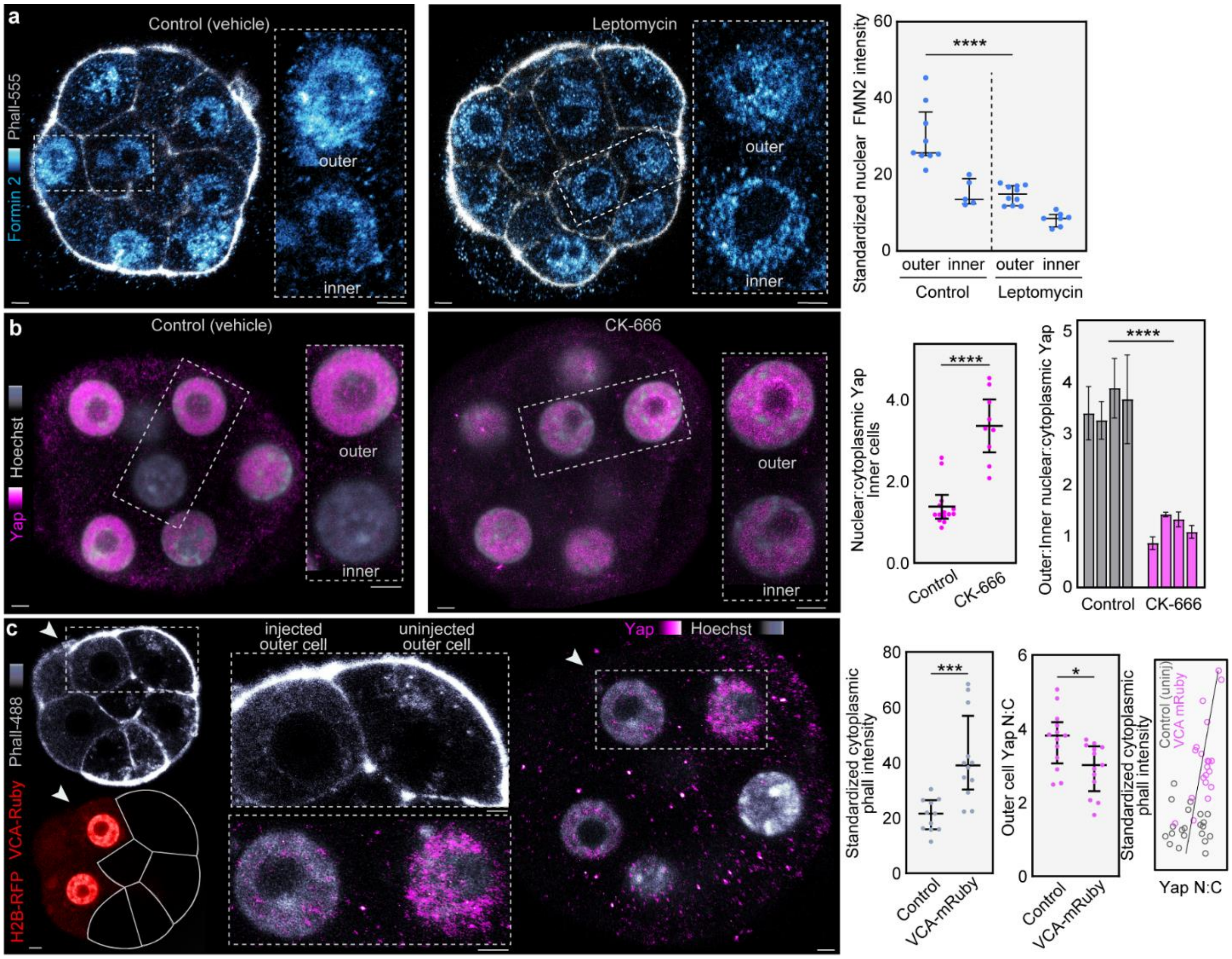

Supplementary Fig 6. Role of Arp2/3 in actin organization

a, Disruption of nuclear transport using leptomycin B reduces levels of nuclear FMN2, as shown using immunofluorescence. n=9 for control outer, n=5 for control inner, n=10 for leptomycin B outer, n=7 for leptomycin B inner. ****P<0.0001, Kruskal-Wallis test. **b**, Treatment with CK-666, a small molecule inhibitor of Arp2/3 complexes, reduces the outer to inner cell nuclear to cytoplasmic ratio of Yap. Insets highlight the differences between nuclear Yap intensity comparing outer to inner cells. Bar graph shows the ratio of nuclear to cytoplasmic Yap between outer to inner cells. Each bar represents one embryo, errors bars represent SD. ****P<0.0001, Mann-Whitney U test comparing group means. **c**, Expression of VCA-Ruby, a conserved Arp2/3 activating domain of WASP proteins, causes increased actin levels and reduced Yap nuclear localization in outer cells. VCA-Ruby and H2B-RFP RNA were microinjected into a single cell of the 2-cell embryo, fixed at the 16-cell stage, stained with phalloidin and immunostained for Yap. Injected cells can be identified by H2B-RFP expression in the nucleus and VCA-Ruby in the cytoplasm (arrowheads). n = 11 for control (uninjected outer cells), n=12 for VCA-Ruby (injected outer cells), ***P=0.0001, *P=0.014, Mann-Whitney U test. All statistical tests are two-tailed. Bars in dot plots represent median and interquartile range. Scale bars, 10 µm.

Supplementary Table 1. Antibody details

Primary Antibody	Supplier	Catalog No.	Dilution
Mouse monoclonal anti-Lamin-A/C (E-1)	SCBT	sc-376248	1:200
Rabbit polyclonal anti-phospho-myosin II	Cell Signaling	3671P	1:200
Rat monoclonal anti-Keratin 8	DSHB	TROMA-I	1:20
Mouse monoclonal anti-α-tubulin	Sigma	T6199	1:500
Rabbit polyclonal anti-Lamin-B1	Protein Tech	12987-1-AP	1:200
Rabbit polyclonal anti-Phospho-Lamin-A/C (Ser22)	Cell Signaling	2026	1:200
Rabbit monoclonal anti-Phospho-Yap	Cell Signaling	13008	1:200
Rabbit polyclonal anti-Cdx2	Abcam	88129	1:200
Rabbit monoclonal anti-Yap	Cell Signaling	84185	1:200
Rabbit anti-Arnot	Gift from H. Sasaki		1:200
Rabbit polyclonal anti-Formin 2	Invitrogen	PA5-65632	1:200

Secondary Antibody	Dye	Catalog No.	Dilution
Goat anti-Mouse IgG Cross-Adsorbed Secondary Antibody	Alexa Fluor 488	A-11001	1:500
Goat anti-Rat IgG Cross-Adsorbed Secondary Antibody	Alexa Fluor 488	A-11006	1:500
Goat anti-Rabbit IgG Highly Cross-Adsorbed Secondary Antibody	Alexa Fluor 488	A-11034	1:500
Goat anti-Mouse IgG Highly Cross-Adsorbed Secondary Antibody	Alexa Fluor 647	A21247	1:500
Goat anti-Rat IgG Cross-Adsorbed Secondary Antibody	Alexa Fluor 647	A-21247	1:500
Goat anti-Rabbit IgG Cross-Adsorbed Secondary Antibody	Alexa Fluor 647	A-21244	1:500

Invitrogen was the supplier for all secondary antibodies.
