# Supplementary material for: The nuclear lamina couples mechanical forces to cell fate in the preimplantation embryo via actin organization
Source: Nat Commun. 2023 May 29;14:3101. doi: 10.1038/s41467-023-38770-5 (PMC10226985; doi:10.1038/s41467-023-38770-5)
Supplement: Supplementary file 3 — Description of Additional Supplementary Files [file 41467_2023_38770_MOESM3_ESM.pdf]

Description of Additional Supplementary Files

File Name: Supplementary Movie 1
Description: Embryos expressing Utrophin-GFP are shown at the 4-cell and 8-cell stages. Expression of Utrophin-GFP, 3D segmentation and quantification of local curvature. Divisions at the 4-cell stage produce higher deformation of cell shape.
