# Supplementary material for: The nuclear lamina couples mechanical forces to cell fate in the preimplantation embryo via actin organization
Source: Nat Commun. 2023 May 29;14:3101. doi: 10.1038/s41467-023-38770-5 (PMC10226985; doi:10.1038/s41467-023-38770-5)
Supplement: Supplementary file 5 — Reporting Summary [file 41467_2023_38770_MOESM5_ESM.pdf]

nature portfolio

Corresponding author(s): Nicolas Plachta, Denny Sakkas

Last updated by author(s): Apr 24, 2023

Reporting Summary

Nature Portfolio wishes to improve the reproducibility of the work that we publish. This form provides structure for consistency and transparency in reporting. For further information on Nature Portfolio policies, see our Editorial Policies and the Editorial Policy Checklist.

Statistics

For all statistical analyses, confirm that the following items are present in the figure legend, table legend, main text, or Methods section.

n/a	Confirmed	
☐	☒	The exact sample size (*n*) for each experimental group/condition, given as a discrete number and unit of measurement
☐	☒	A statement on whether measurements were taken from distinct samples or whether the same sample was measured repeatedly
☐	☒	The statistical test(s) used AND whether they are one- or two-sided<br>*Only common tests should be described solely by name; describe more complex techniques in the Methods section.*
☒	☐	A description of all covariates tested
☐	☒	A description of any assumptions or corrections, such as tests of normality and adjustment for multiple comparisons
☐	☒	A full description of the statistical parameters including central tendency (e.g. means) or other basic estimates (e.g. regression coefficient) AND variation (e.g. standard deviation) or associated estimates of uncertainty (e.g. confidence intervals)
☒	☐	For null hypothesis testing, the test statistic (e.g. *F*, *t*, *r*) with confidence intervals, effect sizes, degrees of freedom and *P* value noted<br>*Give P values as exact values whenever suitable.*
☒	☐	For Bayesian analysis, information on the choice of priors and Markov chain Monte Carlo settings
☒	☐	For hierarchical and complex designs, identification of the appropriate level for tests and full reporting of outcomes
☒	☐	Estimates of effect sizes (e.g. Cohen's *d*, Pearson's *r*), indicating how they were calculated

Our web collection on statistics for biologists contains articles on many of the points above.

Software and code

Policy information about availability of computer code

Data collection	Time-lapse imaging of live samples and single confocal scans af fixed immunostained samples were both performed using a Leica SP8 (mouse embryos) or Nikon A1RHD25 (human embryos).
Data analysis	Image analyses were performed using Imaris 8.2 or 9.7 (Bitplane AG), ImageJ-Win64 Fiji and MATLAB (Version R2018a). Statistical analyses were performed using GraphPad Prism (Version 8.3) and Microsoft Excel (Version 16.3).

For manuscripts utilizing custom algorithms or software that are central to the research but not yet described in published literature, software must be made available to editors and reviewers. We strongly encourage code deposition in a community repository (e.g. GitHub). See the Nature Portfolio guidelines for submitting code & software for further information.

Data

Policy information about availability of data

All manuscripts must include a data availability statement. This statement should provide the following information, where applicable:

- Accession codes, unique identifiers, or web links for publicly available datasets
- A description of any restrictions on data availability
- For clinical datasets or third party data, please ensure that the statement adheres to our policy

All data supporting the findings of this study are available within the paper and its Supplementary Information.

Research involving human participants, their data, or biological material

Policy information about studies with human participants or human data. See also policy information about sex, gender (identity/presentation), and sexual orientation and race, ethnicity and racism.

Reporting on sex and gender	Donated human embryos were de-identified according to the New England Institutional Review Board and preimplantation genetic testing results, including sex, were unknown.
Reporting on race, ethnicity, or other socially relevant groupings	Race, ethnicity or other socially relevant groupings were not considered.
Population characteristics	Donated human embryos were de-identified according to the New England Institutional Review Board, therefore specific characteristics are unknown. In general our IVF females range between 18-43 years of age.
Recruitment	Individuals with embryos who were choosing to discard embryos because of previous successful pregnancies and/or selecting to not pursue further IVF treatment are sent a yearly disposition form about their frozen embryos.<br>(see https://www.bostonivf.com/content/editor/Discard-Frozen-Embryos-Consent-New.pdf).<br>Patients then complete their disposition form to indicate whether they will continue storage, discard embryos or donate to research. Alternatives included ongoing storage versus discarding samples.
Ethics oversight	New England Institutional Review Board

Note that full information on the approval of the study protocol must also be provided in the manuscript.

Field-specific reporting

Please select the one below that is the best fit for your research. If you are not sure, read the appropriate sections before making your selection.

☒ Life sciences ☐ Behavioural & social sciences ☐ Ecological, evolutionary & environmental sciences

For a reference copy of the document with all sections, see nature.com/documents/nr-reporting-summary-flat.pdf

Life sciences study design

All studies must disclose on these points even when the disclosure is negative.

Sample size	No statistical test was performed to determine sample size. Sample size was determined based on prior experience and typical ranges used by research groups in the preimplantation mouse embryo field, and also in accordance to statistical test requirements. Previous work utilizing similar sample sizes includes Zenker et al. 2018 Cell 173:776-791; Zenker et al. 2017 Science 357: 925-928; White et al. 2016 Cell 165(1): 75-87; Lim et al. 2020 Nature 585(7825):404-409
Data exclusions	Embryos excluded from analyses include: 1) Unsuccessfully microinjected embryos that display low or undetectable fluorescence labeling nsuitable for quantitative analysis, and 2) 10-15% of embryos that display arrested or slower development in culture conditions. These exclusion criteria have been utilized in our previous work.
Replication	All experiments in this study were successfully performed at least 3 times with different batches of embryos, mRNA or siRNA preparations.
Randomization	Embryos were randomly allocated into experimental groups. All embryos and cells within embryos were randomly selected for analysis.
Blinding	Successfully developed and imaged embryos have to be selected for subsequent analysis, thus investigators were not blinded to group allocation during data collection. However, only embryos that displayed appropriate morphologic development prior to treatment were included thus introducing minimal bias during embryo processing. Investigators were blinded for computational analysis following acquisition of imaging data.

Reporting for specific materials, systems and methods

We require information from authors about some types of materials, experimental systems and methods used in many studies. Here, indicate whether each material, system or method listed is relevant to your study. If you are not sure if a list item applies to your research, read the appropriate section before selecting a response.

Materials & experimental systems

n/a	Involved in the study	
☐	☒	Antibodies
☒	☐	Eukaryotic cell lines
☒	☐	Palaeontology and archaeology
☐	☒	Animals and other organisms
☒	☐	Clinical data
☒	☐	Dual use research of concern
☒	☐	Plants

Methods

n/a	Involved in the study	
☒	☐	ChIP-seq
☒	☐	Flow cytometry
☒	☐	MRI-based neuroimaging

Antibodies

Antibodies used

Mouse monoclonal anti-Lamin-A/C (E-1) SCBT sc-376248 1:200
Rabbit polyclonal anti-phospho-myosin II Cell Signaling 3671P 1:200
Rat monoclonal anti-Keratin 8 DSHB TROMA-I 1:20
Mouse monoclonal anti-α-tubulin Sigma T6199 1:500
Rabbit polyclonal anti-Lamin-B1 Protein Tech 12987-1-AP 1:200
Rabbit polyclonal anti-Phospho-Lamin-A/C (Ser22) Cell Signaling 2026 1:200
Rabbit monoclonal anti-Phospho-Yap Cell Signaling 13008 1:200
Rabbit polyclonal anti-Cdx2 Abcam 88129 1:200
Rabbit monoclonal anti-Yap Cell Signaling 84185 1:200
Rabbit anti-Arnot Gift from H. Sasaki 1:200
Rabbit polyclonal anti-Formin 2 Invitrogen PA5-65632 1:200

Goat anti-Mouse IgG Cross-Adsorbed Secondary Antibody Alexa Fluor 488 A-11001 1:500
Goat anti-Rat IgG Cross-Adsorbed Secondary Antibody Alexa Fluor 488 A-11006 1:500
Goat anti-Rabbit IgG Highly Cross-Adsorbed Secondary Antibody Alexa Fluor 488 A-11034 1:500
Goat anti-Mouse IgG Highly Cross-Adsorbed Secondary Antibody Alexa Fluor 647 A21247 1:500
Goat anti-Rat IgG Cross-Adsorbed Secondary Antibody Alexa Fluor 647 A-21247 1:500
Goat anti-Rabbit IgG Cross-Adsorbed Secondary Antibody Alexa Fluor 647 A-21244 1:500

Invitrogen was the supplier for all secondary antibodies.

Validation

All antibodies were previously validated by vendors and/or published work. Relevant studies include:

Keratin 8: filamentous keratin network in ES cells (Schwarz et al. 2015 Sci. Rep. 5: 9007); keratin network in mouse blastocysts showing trophectoderm-specific localization (Ralston and Rossant 2008 Dev. Biol.). Specific staining in mouse and human embryos (Lim et al., 2020 Nature)
Lamin-A/C: Specific staining in mouse embryos (Vergnes et al., 2004 PNAS)
Lamin-Bl: Specific staining in mouse oocytes (Fei et al., 2022 Environmental Toxicology)
Phospo-Lamin-A/C (Ser22): Nucleoplasm localization in HeLa cells (Kochin et al., 2014 J Cell Sci)
Phospho Myosin II: Localization in mouse mammary glands (Schipper et al., 2019 Nat. Comm)
Phospho-Yap (127): IHC localization in colonic epithelium (Georgescu et al., 2016 Neoplasia)
Yap: Specific staining of nuclei of polar outer cells of the mouse embryo (Anani et al. 2014 Development)
Cdx2: Specific staining of nuclei in mouse embryos throughout preimplantation development, and elevated levels in trophectoderm cells of the blastocyst relative to those of the inner cell mass (White et al. 2016 Cell); specific staining of nuclei in morula-stage embryos (Samarage et al. 2015 Dev. Cell)
Arnot: Localization in preimplantation mouse embryos (Hirate et al., 2013 Curr. Biol)
α-tubulin: Specific localization to interphase and cytokinetic microtubule bridges in mouse embryos (Zenker et al. 2017 Science); specific localization to mitotic spindles in mouse embryos (Zenker et al. 2018 Cell).
Formin-2: Localization in human cell line BJ to plasma membrane and actin filaments (Invitrogen testing data)

Animals and other research organisms

Policy information about studies involving animals; ARRIVE guidelines recommended for reporting animal research, and Sex and Gender in Research

Laboratory animals

Mouse strains included 2 inbred (FVB/NTac and C57BL/6JInv) and 2 outbred (Hsd:NSA(CF-1) and IcrTac:ICR) animal lines purchased from approved vendors (i.e. Charles Rivers Laboratories, Envigo and Jackson Laboratory). All mice were maintained within a BSL2 animal facility at the University of Pennsylvania in pathogen-free conditions with access to water and food ad libitum, a 12-hour dark / 12-hour light cycle between 07:00 and 19:00 in a temperature (68°F - 76°F) and humidity (30% - 70%) controlled room. Female mice at 8 weeks of age, were superovulated using 5 IU of pregnant mare serum (PMS, National Hormone and Peptide Program) gonadotropin given intraperitoneally and 5 IU of recombinant chorionic gonadotrophin (CG, National Hormone and Peptide Program) given 48 h after and immediately before mating, according to animal ethics guidelines of the University of Pennsylvania IACUC committee.

Wild animals

No wild animals were used.

Reporting on sex	Embryo sex was not determined and therefore not considered during analysis.
Field-collected samples	No field-collected samples were used.
Ethics oversight	All mouse work was reviewed and approved by the .Institutional Animal Care and Use Committee of the University of Pennsylvania (Protocol #806983)

Note that full information on the approval of the study protocol must also be provided in the manuscript.
